# Economic burden of non-communicable diseases on households in Nigeria: evidence from the Nigeria living standard survey 2018-19

**DOI:** 10.1186/s12889-023-16498-7

**Published:** 2023-08-17

**Authors:** Adelakun Odunyemi, Taslima Rahman, Khurshid Alam

**Affiliations:** 1 Murdoch Business School, Murdoch University, 90 South Street, Perth, WA 6150 Australia; 2 Hospitals’ Management Board, Akure, Ondo State Nigeria; 3 Murdoch Business School, Murdoch University, Perth, Western Australia Australia; 4 Institute of Health Economics, University of Dhaka, Dhaka, 1000 Bangladesh

**Keywords:** Non-communicable diseases, Out-of-pocket health expenditure, Catastrophic health expenditures, Poverty, Productivity loss

## Abstract

**Background:**

The importance of non-communicable diseases (NCDs) in Nigeria is reflected in their growing burden that is fast overtaking that of infectious diseases. As most NCD care is paid for through out-of-pocket (OOP) expenses, and NCDs tend to cause substantial income losses through chronic disabilities, the rising NCD-related health burden may also be economically detrimental. Given the lack of updated national-level evidence on the economic burden of NCDs in Nigeria, this study aims to produce new evidence on the extent of financial hardship experienced by households with NCDs in Nigeria due to OOP expenditure and productivity loss.

**Methods:**

This study analysed cross-sectional data from the most recent round (2018–19) of the Nigeria Living Standard Survey (NLSS). Household-level health and consumption data were used to estimate catastrophic health expenditure (CHE) and impoverishing effects due to OOP health spending, using a more equitable method recently developed by the World Health Organization European region in 2018. The productivity loss by individuals with NCDs was also estimated from income and work-time loss data, applying the input-based human capital approach.

**Results:**

On average, a household with NCDs spent ₦ 122,313.60 or $ 398.52 per year on NCD care, representing 24% of household food expenditure. The study found that OOP on cancer treatment, mental problems, and renal diseases significantly contribute to the cost of NCD care. The OOP expenditure led to catastrophic and impoverishing outcomes for households. The estimations showed that about 30% of households with NCDs experienced CHE in 2018, using the WHO Europe method at the 40% threshold. The study also found that the cost of NCD medications was a significant driver of CHE among NCD-affected households. The results showed heterogeneity in CHE and impoverishment across states and geographical regions in Nigeria, with a higher concentration in rural and North East geopolitical locations. The study also found that 20% of NCD-affected households were impoverished or further impoverished by OOP payment, and another 10% were on the verge of impoverishment. The results showed a negligible rate of unmet needs among households with NCDs.

**Conclusions:**

The study highlights the significant effect of NCDs on Nigerian households and the need for effective policy interventions to address this challenge, particularly among the poor and vulnerable.

**Supplementary Information:**

The online version contains supplementary material available at 10.1186/s12889-023-16498-7.

## Background

Non-communicable diseases (NCDs) have caught the attention of the global health community because they are responsible for about 41 million deaths a year, equivalent to 71% of all deaths globally [1]. Of the fatalities caused by NCDs, 17.9 million are attributable to cardiovascular diseases (CVDs), 9.3 million to cancer, 4.1 million to respiratory diseases, 1.5 million to diabetes, and the remaining 8.2 million to other NCDs [1]. About 47% of deaths from NCDs are premature, and most of the deaths (31.4 million) occur in low- and middle-income countries (LMICs), which now face a double burden of infectious diseases and NCDs. Similarly, NCDs are increasingly pushing the mortality figures in Sub-Saharan Africa (SSA). According to the projection of the World Bank, mortality from NCDs in Africa will overtake infectious diseases by 2030 [2]. Aside from being accountable for premature mortality, NCDs inflict considerable loss through chronic disabilities [3]. The number of disability-adjusted life years (DALYs) lost to NCDs is on the rise in SSA, while that for the composite of communicable, maternal, neonatal, and nutritional disorders (CMNND) is plummeting [4]. In 2019, NCDs were responsible for Africa’s DALY loss of 167 million, compared to 164 million for infectious diseases.

Like other SSA countries, Nigeria is undergoing an epidemiological transition with a rising burden of NCDs. Nearly 30% of all deaths in Nigeria are due to NCDs. The risk of premature death from cardiovascular diseases (CVDs), cancers, respiratory diseases, and diabetes among Nigeria’s 30 to 69-year-olds is 22% [1]. The DALYs lost to NCDs skyrocketed by about 21.3% from 24,987.4 in 2010 to 30,306.5 in 2019. During the same period, the DALY loss to infectious diseases decreased by roughly 6.5% [5]. If this trend continues, the DALYs lost to NCDs will soon surpass those of infectious diseases.

A crucial burden not reflected by the figures above is households’ financial hardship from out-of-pocket (OOP) healthcare expenditure on NCDs and productivity loss from hospitalisation. The latest available Nigeria’s National Health Accounts show that of the ₦384.4 billion (US$1.26 billion) and ₦374 billion (US$1.22 billion) spent, respectively, on NCDs and Human Immune-deficiency Virus (HIV) in Nigeria in 2017, public expenditure was 63.4% for HIV and only 25.6% for NCDs. Thus, households with NCDs in Nigeria spent a preponderance of 74.4% of current health expenditure on NCDs as OOP [6].

NCDs usually incur productivity loss due to hospitalisation, chronic disability, and death [3]. Studies have revealed that these costs could be substantial, deepening families into financial difficulties [7]. Nigeria bore about 50.9% of the loss of productivity due to illness in LMICs, and almost 78% of the Economic Community of West African States (ECOWAS) total GDP loss, and approximately 40% of this was due to NCDs [8]. These economic consequences from OOP expenditure and productivity loss from NCDs cumulatively contribute to poverty in Nigeria [9].

The burden of household OOP healthcare expenditure is measured by two indicators of financial protection (FP): catastrophic health expenditure (CHE) and impoverishing health spending [10]. A household incurs CHE if its OOP healthcare expenditure exceeds some pre-defined threshold of its total income or consumption expenditure, affecting its welfare through cutbacks on essential consumption (such as food). Households are impoverished (or further impoverished) due to OOP when such expenses push them below (or further down) the poverty line [11]. OOP payment for NCD care predisposes households to CHE and drives many into impoverishment [12]. It has been demonstrated that NCDs-related OOP health expenditure is potentially catastrophic and impoverishing in Southwestern Nigeria [13]. There are two critical issues in the methodological approaches to estimating the economic burden of NCDs. First, older methods, such as the budget share (Wagstaff & Doorslaer (2003) [14]) actual food expenditure, WHO capacity-to-pay/partial normative food expenditure methods (Xu et al. (2003) [15] and WHO (2005) [16]), used in financial protection estimations are fraught with equity and policy-related problems [17, 18]. Second, though they may contribute less to mortality figures, some often-neglected NCDs in the estimation have been shown to cause substantial productivity losses through chronic disabilities [19]. For example, while DALY loss for each major NCD decreased or plateaued between 1999 and 2010, mental, musculoskeletal, and endocrine diseases rose [20]. In Nigeria, cancer DALY loss (2595.0) was less than gastroenterological diseases (3025.5) in 2019. Diabetes DALY loss was about two-thirds of haematological disorders. Without considering these often-forgotten NCDs, results on the economic burden of NCDs may be inaccurate. In SSA, there is a shortage of quality research on the economic burden of diseases [21], especially for NCDs [7, 22, 23].

In Nigeria, the few studies addressing the economic burden of NCDs are limited regarding the population sampled, data recency, and the number of NCDs covered [24–27]. Productivity loss was only included in two of these studies [13, 25]. A recent multicountry analysis of the household economic burden of NCDs excluded Nigeria because of data unavailability [28]. Bridging the above gap in the literature, we seek to estimate the extent of the financial hardship experienced by households in Nigeria because of OOP expenditure and productivity loss from NCDs. Thus, the findings from our study would expand the literature on the economic burden of diseases in Nigeria and contribute to policy design to lower the financial burden of NCDs in Nigeria.

## Methods

### Data

We conducted a descriptive study using nationally representative secondary cross-sectional data from the most recent round of the Nigerian Living Standard Survey (NLSS) conducted by Nigeria’s National Bureau of Statistics (NBS) between September 2018 and September 2019 [29]. The survey used a stratified multi-stage random sampling to sample 22,200 households with a mean household size of 5.06 and about 116,320 individuals. It covered 600 households per state, including the Federal Capital Territory (FCT), with a 95% response rate. The survey collected individual and household level data, including demographic variables, health problems and access, education, labour, consumption, housing, and assets. We excluded Borno State from our analyses because insecurity prevented the survey of many households in the state.

### Diseases and health conditions classification

The health conditions provided in the NLSS 2018/19 were self-reported. Respondents were asked: “Were you sick or injured in the last 30 days?” Those who answered “yes” were asked to list two illnesses/injuries in order of severity. Unlisted conditions were entered under “other (specify)”.

We matched the two lists of health conditions and the “other (specify)” category to ICD-10 classifications to distinguish between NCDs (excluding injuries) and infectious diseases. Using the list provided in the WHO (2013) report [30] and the Gouda et al. (2019) study [4], we arrived at 14 NCD categories: CVDs, cancers (neoplasms), respiratory diseases, diabetes, mental disorders, neurological diseases, haematological diseases (mainly sickle cell diseases), sense organ diseases, renal diseases (kidney), gastroenterological (digestive) diseases, musculoskeletal diseases, dermatological diseases (skin and subcutaneous), dental (oral) diseases and Other NCDs (urinary disorders, gynaecological diseases, and endocrine disorders) (Supplementary Table 1). We grouped injuries under “other illnesses and injuries” because they are not NCDs.

Depending on how they were named in the survey, some NCDs may belong to “other illnesses and injuries” and vice versa. For example, the survey lumped all body pains together, making chronic musculoskeletal pains indistinguishable. Also, for cough, the primary disease conditions, such as COPD, were not specified. Thus, we omitted body pain and cough to reduce bias. Diseases under the names respiratory, tooth, ear, and eye problems in the survey may be either NCDs or infectious diseases. So, they were excluded. The age group covered in our estimations was from five years and above. We excluded the under-five age group to avoid misclassifying infectious diseases commonly found in under-five children in these ambiguous categories [25]. This would not likely underestimate the number of NCDs in our study since the peak age for NCDs in Nigerian children was 6–11 [31], and many children with NCDs don’t live to age 5 in LMICs [32].

### Health services utilisation and cost

Respondents that utilised healthcare were asked about the duration of the illness/injury and the amount paid for consultation (excluding drugs). They were further asked about any drugs purchased. The recall period for both was 30 days. In addition, they were asked if they had been admitted to a hospital or health facility in the previous 12 months. Those that answered in the affirmative were asked how long and how much they paid (excluding drugs). Following Mahal et al. (2010) [33], the costs of consultation and drugs were annualised by multiplying them by 12.17, presuming that the visits were uniformly distributed throughout the year. Individual-level data were collapsed and merged with household-level data for household-level estimates.

### Consumption and income

We aggregated the consumption data in the survey at the household level. They covered five types of household consumption: food (bought, self-produced, and gifted), non-food (various regularly purchased commodities and services), education, health, and rent. Before statistical analyses, household income and consumption data were examined for missing values, and none was found. Unrealistic negative or no food expenditures data were dropped.

### Analytical strategy and statistical analysis

#### Estimation of household catastrophic health expenditure

Wagstaff and Doorslaer’s budget share [14] and WHO’s capacity-to-pay (CTP) [15] are methods commonly used to estimate CHE. Both methods use OOP health spending as the numerator. Typically, OOP excludes third-party reimbursements and insurance payments. The denominator in the budget-share method is household income or consumption, while it is CTP in the WHO CTP method. CTP is the amount left for a household to spend after basic (or subsistence) needs are met. For a household, $i$, with total consumption expenditure ${ THC}_{exp }$, its CTP is expressed as:

1 $${CTP}_{i}={ THC}_{exp }- {SE}_{i}$$

where ${SE}_{i}$ stands for expenditure on subsistence (basic) needs, which is the minimum requirement to maintain basic life in a society. ${SE}_{i}$ is calculated from a defined basic need line, ${Pl}_{n}$ and the equivalent household size, ${eqsize}_{i}$(which adjusts for household size and composition according to the economy of scale in consumption needs), using the equation:

2 $${SE}_{i}={Pl}_{n}\times {eqsize}_{i}$$

The original WHO CTP method, known as the WHO standard method, is the most frequently used method in studies in Nigeria [34]. The WHO European regional office recently pioneered an improved CTP method called the WHO-Europe method [10]. The WHO standard method is partially normative because it recognises only food as a basic need. The WHO-Europe method is fully normative, including other basic needs such as housing and utilities (water, electricity, cooking gas and other fuels for heating) [10]. Conventionally, restaurant food, tobacco, and alcohol are not considered basic needs [16].

The WHO-Europe method differs from the WHO standard method in three main ways. First, it uses a poverty line which reflects spending on fully normative basic needs among relatively poor households (those between the 25th and 35th percentiles of the household consumption distribution) in a country [10]. The WHO standard method, however, uses food expenditure among households in the average consumption distribution (the 45th and 55th percentiles) [15, 35]. Second, unlike the WHO standard method, the WHO-Europe method allows very poor households with total household consumption expenditure below the subsistence line to have negative CTP [10]. Instead, the latter substitutes actual food expenditure for the higher SE [16]. Third, the WHO-Europe method uses the OECD equivalence scale [10, 36] instead of the WHO standard method’s scale [15].

The WHO Europe method provides the benefit of using a basic need line closest to national poverty lines [10]. Moreover, it is more equitable than the budget-share and WHO standard methods, which are pro-rich, reducing CHE among the poor [17]. The budget share method, adopted as the official FP monitoring indicator for SDG 3.8.2, is worse off in this respect [10, 35]. WHO Europe method’s proponents assented to its broader applicability in high- and middle-income countries [10]. Studies from Nigeria and other LMICs, such as India and Bangladesh that used similar methods attest to this fact [21, 37–39].

Given the weaknesses of the other methods, we leveraged the WHO-Europe method to produce actionable evidence of financial hardship from NCDs in Nigeria [10, 17]. We adapted this method for Nigeria in two ways. First, electricity and heating fuel were removed from the list of basic utilities, leaving cooking fuel. House heating is not a basic need in Nigeria, and only 59% of households have electricity [40]. Second, clothing was introduced because it was adjudged a necessity [37]. As recommended in the original WHO-Europe method [10, 17], we only included actual rent in our calculations.

The WHO-Europe and WHO standard thresholds are 40%, and the budget share threshold is 10% [41]. Thresholds are arbitrary. Rashidian et al. (2018) [42], using the Receiver Operator Characteristic (ROC) curve and Kappa method, computed 20% and 35% as the optimal thresholds for the budget share and CTP methods, respectively. Using different thresholds by researchers produces very different rankings among countries, impeding global monitoring [41]. For sensitivity purposes, following Rashidian et al. (2018) [42], we varied the thresholds used: 20%, 25%, 30%, 35%, and 40%. For comparison with other studies, we have included CHE estimates at different thresholds for the budget share and WHO standard methods (Supplementary Table 2).

#### Estimation of household impoverishment

Household impoverishing health expenditure occurs if a household is impoverished by OOP health spending. Households are classified into five categories based on their remaining consumption expenditure relative to the poverty line after OOP health expenses [10, 35]. We used Nigeria’s 2018 national poverty line of ₦137,430 (US$447.78) [43]. A household is “impoverished” when its total consumption, though above the poverty line before OOP health spending, falls below the poverty line after OOP spending. A household whose total consumption was already below the poverty line before incurring OOP health payments is said to be “further impoverished”. “At-risk of impoverishment” and “not-at-risk of impoverishment” households have total consumption above the poverty line before or after OOP health spending. A household’s total consumption relative to 120% of the poverty line after OOP health payments determines whether it is “at risk of impoverishment” or not. Households whose total consumption falls on or below this 120%-line (but above the poverty line) are “at risk of impoverishment”, and those who remain above it after OOP spending are “not at risk of impoverishment”. The “non spender” group had no OOP spending. They could fall above or below the poverty line. This group includes, among others, those who missed care due to cost [35].

WHO-Europe method highlights three important categories of households obscured in the traditional impoverishment measurements [44]. These groups include the “further impoverished”, “at risk of impoverishment”, and “non-spenders”. The first two are vulnerable groups as crucial as the impoverished category []. Not considering the “further impoverished” suggests OOP health expenditures are only harmful if they cause poverty, not if they worsen it. Also, the “at risk of impoverishment” group is critical in poverty-prevention policies [10]. A high percentage of the “non-spender” group indicates significant forgone care in a population [35]. In this case, low CHE estimates could give a false impression that the population enjoys FP [10]. Forgone care worsens health conditions, lowering the household’s productivity and welfare [12].

#### Estimation of productivity loss

Household productivity loss was estimated using the methods suggested in the WHO Global TB Programme protocol [18]. We used an input-based human capital approach to calculate productivity loss for a household, $i$ with $n$ members:

3 $$\sum _{i}^{n}{P}_{loss}= \sum _{i}^{n}{(t}_{confinement}+{t}_{missed})\ast {w}_{total}$$

Where, ${P}_{loss}$ is the total annual productivity loss of an individual in the household, ${t}_{confinement}$ is the number of days the individual is confined to a hospital bed, ${t}_{missed}$ is the number of days the individual missed from primary activities, (apart from days confined to a hospital bed), ${w}_{total}$ is the daily income of an individual and the sum of the time, ${(t}_{confinement}+{t}_{missed})$ is the total work-time lost.

The number of days individuals missed from their usual activity and the number of days confined to bed had recall periods of 30 days and 12 months, respectively, in the survey. The former was annualised by multiplying by 12.17 [33].

Our productivity loss estimation is from ill health, not death and relates to personal but not employer-related losses. We assumed there was constant income, no payment for sick-off periods, no social safety nets or health insurance and no compensatory input from other household members. Nigeria has less than 4% health insurance coverage [42] and little social protection against illness [45]. Also, we assumed an informal caregiver’s income loss is equal to the patient’s. This may not be true for informal and seasonal workers.

The debate in the human capital approach for productivity loss calculation has been about the appropriateness of income used [46, 47]. In this study, we employed three approaches proposed by the WHO [44] to determine our income values. First, respondents’ pre-illness incomes were used. We imputed for missing data the average income of people in their income quintile. This approach has, however, been blamed for equity issues [18, 46, 47]. Multiplying the unadjusted productivity loss by the sample’s labour force participation rate, 72.21% (95% CI: 71.85–72.58), corrected for unemployment. Second, we used the lowest-paid unskilled government worker pay, $30,000 (US$9775), in 2018 [48]. Third, we used Nigeria’s annual GDP per capita of ₦ 622,372.38 (US$2027.8) in 2018 [49] instead of the minimum wage [47]. We accounted for unemployment in the last two methods using half the general wage as suggested by WHO (2015) [18]. The three methods jointly reduce errors arising from imputed income, improving the spread and sensitivity of our estimates.

Outliers (about 0.05%) in our dataset were identified and removed before all analyses, using the trimming method. We estimated the OOP expenditure, CHE and impoverishment at the household level. The sampling weight supplied with the data was used in all our analyses for national representativeness. Data analysis was performed using Stata MP, version 16.0 (StataCorp, Texas, USA).

## Results

The prevalence of NCDs in the sample was 16.80% (95% CI: 16.40–17.20), and comorbidity was 4.30% (95% CI: 3.87–4.93) (Supplementary Table 3). Although males and females in the sample were almost equal (49.32% and 50.68%), more women reported NCDs (61.00%; 95% CI: 59.72–62.26). In rural regions, NCD prevalence was greater (74.83%) (Supplementary Table 3). Most common NCDs were gastroenterological (51.69%), cardiovascular (15.00%), and sense organ (9.70%) (Supplementary Table 3).

There were 4,560 households (21.00%) affected by NCDs. NCDs were more prevalent in the middle-class (21.31%; 95% CI: 19.99–22.70%) and poorest (21.01%; 95% CI: 19.35–22.76%) households than in the richest (16.29%; 95%CI: 14.50–18.25) households (Supplementary Table 4).

A household with NCDs spent an annual average of ₦122313.60 (95% CI: 111005.70–133621.50) [$398.52 (95% CI: 361.68–435.36)] as OOP, which was about 24% of household food expenditure (Supplementary Table 5). Their annual per capita OOP expenditure was 20092.44 (95% CI: 18158.81–22026.08) [$65.67 (95% CI: 59.17–71.77](Supplementary Table 6). This was about 18% of the country’s annual basic minimum income of ₦360,000 [$1172.94]. The mean OOP expenditure by NCD-affected households was 93.94% of total household health expenditure (Supplementary Table 6). Those who utilised private health facilities spent about 1.4 times more than public facilities (Supplementary Table 7). People with NCD spent more significantly on drugs than outpatient and inpatient care (Supplementary Figure 1). Cancer, renal disease, and mental problems dominated the OOP costs for diseases. Medicines drove OOP costs for malignancies and renal disease, while outpatient consultations drove mental illness (Supplementary Figure 1). Moreover, the richest households had a slightly higher budget share of OOP (13.63%; 95% CI: 10.62–16.64) on NCDs than the poorest households (10.34% 95% CI: 9.56–11.12) (Supplementary Table 8).

Table 1 shows that the overall incidence of CHE from NCDs, using the WHO-Europe method at a 40% threshold, was 28.54% (95% CI: 26.69–30.39), compared with all conditions (including NCDs) (17.06%; 95% CI: 16.07–18.05). The WHO standard and budget share methods showed similarly high NCD-related CHE (Supplementary Table 2). They both produce lower CHE than the WHO-Europe method, but the budget share method concentrated CHE among the rich (Supplementary Table 9). CHE reduces as the threshold used increases in all methods.

**Table 1:** Catastrophic health expenditure at various thresholds in Nigeria, 2018-19

Health conditions	Catastrophic health expenditure at various thresholds (95% CI)^b^				
	20%	25%	30%	35%	40%
**Households with NCDs** ***n*** **[%]**	2,433 [49.75] (47.74–51.76)	2,111 [42.06] (40.05–44.07)	1,849 [36.26] (34.30–38.21)	1,656 [31.98] (30.07–33.89)	1,505 [28.54] (26.69–30.39)
**All households** ^a^ ***n*** **[%]**	6,995 [27.93] (26.72–29.14)	6,086 [23.77] (22.63–24.90)	5,414 [20.77] (19.69–21.85)	4,937 [18.75] (17.75–19.79)	4,567 [17.06] (16.07–18.05)

^a^Estimate for OOP spending on all health conditions, including NCDs and injuries

^b^95% CIs are shown in round parenthesis underneath each estimate of catastrophic health expenditure

Rural households had a higher incidence of CHE from NCDs (34.79%) than urban households (14.97%), and CHE was highly concentrated among the poor (Supplementary Table 10). The incidence of CHE is 81.71% among the poorest households compared to 8.04% among the richest.

OOP spending on medication is the key driver of CHE among households with catastrophic OOP NCD spending, regardless of economic status (Supplementary Table 11). Among NCD-affected households with CHE, 87.32% of OOP was spent on medicine, while it approximates 6% each for inpatient and outpatient care. The poorest NCD-affected households with CHE spent 91.82% of OOP on medicine (8.61% more than the richest households).

**Fig. 1:**
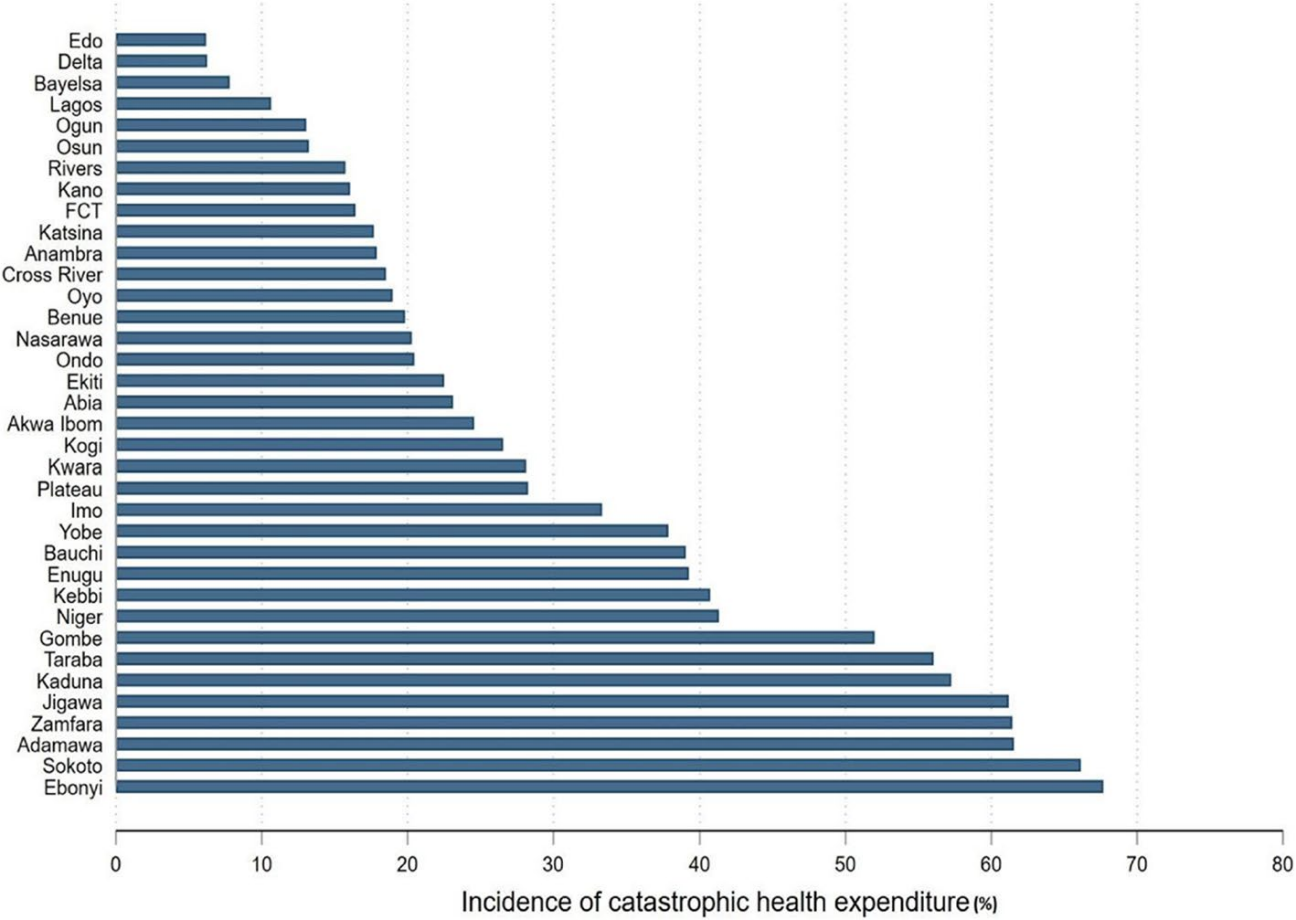
Incidence of Catastrophic Health Expenditure at 40% Threshold from Noncommunicable Diseases across the 35 States and the Federal Capital Territory (FCT) of Nigeria, 2018-19. Notes: Borno State was not included in our analyses because representative data could not be collected due to insurgency

The distribution of NCD-induced CHE by geopolitical zone in Supplementary Table 12 shows the highest [39.40 (95% CI: 37.65–41.15)] and lowest [4.98 (95% CI: 4.27–5.69)] mean CHE in the North East and South West zones, respectively. The disaggregated result by the state in Fig. 1 shows there were more than 60% of NCD-affected households with CHE in Ebonyi, Sokoto, Adamawa, Zamfara, and Jigawa.

About 5.35% (95% CI: 4.73–6.04) of non-poor NCD households were impoverished, compared to 3.17% (95% CI: 2.94–3.41) of all households (Table 2). 12.74% of poor NCD-affected households were further impoverished, compared to 10.68% for all conditions. Also, more NCD households were at risk of impoverishment. NCD-affected households had a lower percentage of non-spenders (2.92%; 95% CI: 2.47–3.45) than all households (23.39%; 95% CI: 22.84–23.97). Sokoto, Jigawa, Ebonyi, and Adamawa lead in NCD-related impoverishment, comparable to CHE. Zamfara and Jigawa had the most impoverished and further impoverished households (approximately 11.11% and 42.35%, respectively) (Supplementary Figure 2).

**Table 2:** Impoverishing effects of OOP health expenditure in Nigeria, 2018-19

Household type	Impoverishment risk categories (%) (95% CI)^b^				
	Non-spender	Not at risk of impoverishment	At risk of impoverishment	Impoverished	Further impoverished
**Households with NCDs** *n* **[%]**	133 [2.92] (2.47–3.45)	3,159 [69.28] (69.92–70.60)	443 [9.71] (8.89–10.61)	244 [5.35] (4.73–6.04)	581 [12.74] (11.80–13.74)
**All households** ^a^ ***n*** **[%]**	5,048 [23.39] (22.84–23.97)	12,076 [55.97] (55.31–56.64)	1,463 [6.78] (6.45–7.13)	684 [3.17] (2.94–3.41)	2,303 [10.68] (10.27–11.09)

^a^Estimate for OOP spending on all health conditions, including NCDs and injuries

^b^95% CIs in round parenthesis underneath each estimate

An individual lost 58.51 (95% CI: 56.30–60.73) productive days per year due to NCDs, compared to 13.74 (95% CI: 13.52–13.96) days for all diseases (Table 3). Aggregated time lost to NCD care was 330,773.70 days (95% CI: 318,248.50–343,298.80) or 906.23 years. Mental disorders and neurological diseases had the most extended hospital stays and missed primary activities (Supplementary Figures 3 and 4).

Table 3 also shows the average and total NCD productivity loss in 2018-19. On average, an individual with NCDs lost ₦36,150.51 (95% CI: 34,142.54–38,158.48) (US$117.79) each year. The annual productivity loss for all NCD-affected people in our sample was at least ₦198 million (95% CI: 179–216 million) (US$0.65 million).

**Table 3:** Annual individual-level productivity loss in Nigeria, 2018-19

Non-communicable diseases	Annual workdays lost (days)	Annual productivity loss (₦)		
	(95% CI)^e^	Method 1^a^ (95% CI)^β^	Method 2^b^ (95% CI)^β^	Method 3^c^ (95% CI)^β^
Mean	58.51 (56.30–60.73)	58571.20 (55587.44–61554.96)	29756.31 (27827.11–31685.51)	20124.02 (19013.07–21234.97)
Total	330773.70 (318248.50–343298.80)	670 million (602–739 million)	323 million (288–358 million)	198 million (179–216 million)
**All conditions** ^d^				
Mean	13.74 (13.52–13.96)	12989.43 (12711.50–13267.36)	9652.78 (9403.03–9902.53)	4842.77 (4720.23–4965.32)
Total	1547103.00 (1522255.00–1571951.00)	1.46 billion (1.43–1.49 billion)	1.09 billion (1.06–1.12 billion)	0.55 billion (0.53–0.69 billion)

Three methods were utilised to calculate income loss.

^a^ *Method 1* uses survey pre-illness income. The average income of people in a similar quintile was used to impute missing income data. The unemployment rate was adjusted using the sample’s labour force participation.

^b^ *Method 2* uses Nigeria’s monthly minimum wage of ₦ 30,000 (US$97.75) for employed and a half for unemployed people.

^c^ *Method 3* utilises Nigeria’s annual GDP per capita of ₦ 622,372.38 (US$2,027.8) instead of the minimum wage for everyone employed and a half for the unemployed.

^d^All conditions denote all illnesses, including non-communicable diseases and injuries.

^e^95% CIs are shown in parentheses underneath each estimate of workdays lost and productivity loss.

## Discussion

Our study represents a comprehensive analysis of Nigeria’s burden of non-communicable diseases (NCDs), utilising the latest nationally representative data and equitable methods. It indicates that households with NCDs are heavily impacted by high OOP expenditure and substantial productivity loss.

On average, an NCD-affected household in our study spent ₦122313.60 or $398.52 per year as OOP, which represents about 24% of household food expenditure or 18.7% of Nigeria’s 2018 GDP per capita [49]. This is in line with other studies that have reported substantial OOP expenditure on NCDs in Nigeria and elsewhere [21, 22, 28]. Some studies in Southwestern Nigeria indicate that average monthly out-of-pocket payments for NCDs range between $46 and $417 [13, 25, 50]. Similarly, Okafor et al. (2022) showed that the direct costs of care for diabetes mellitus and hypertension were more than 25% of the patient’s income [51]. Regarding disease burden, we found that OOP on cancer treatment, mental problems, and renal diseases contributed substantially to the cost of NCD care in Nigeria. This finding is in tandem with studies showing prohibitively high care costs for these chronic conditions in Nigeria [52–55]. This could be over $3889.4 per annum for cancer patients [56]. Given the increased prevalence of NCDs and related risk factors in Nigeria, these expenses substantially influence the country’s economy [57].

It has been shown that OOP expenditure greater than 20% and 29% for an individual and household predisposes them, respectively, to catastrophic and impoverishing outcomes [58, 59]. Since the mean per capita OOP expenditure by NCD-affected households in our study was more than 90% of the total health expenditure, our findings unsurprisingly confirmed the results from those studies. Using the WHO Europe method at the 40% threshold, our estimations showed that about 30% of households with NCDs experience CHE in 2018. Using the same threshold as our study, Janssens et al. (2016), Akintunde et al. (2018), and Ibukun & Adebayo (2021) obtained NCD-related CHE of 8.8%, 12.1% and 20%, respectively [13, 50, 60]. These values are expected to differ from that in our study because of differences in methodological approach, survey time and data collection method. Apart from the surprisingly low CHE in the first study, the estimates from the other two, despite not using nationally representative data, were close to our estimates (13.8%) using the WHO standard method they used.

The slightly higher budget share of OOP among the rich in our study was possibly due to their propensity to use more expensive but less time-wasting private health facilities. Although this private care utilisation by the rich should predispose them to higher CHE than the poor [61], this was not the case, as revealed in our study. The most likely reason was that the share of their health spending to household consumption is generally lower than the poor. Moreover, private facilities typically provide higher quality and efficient care, which reduces frequent and unnecessary health spending. Also, the rich are more likely to engage in preventative efforts to retain good health, reducing their need for regular healthcare use. The shift in CHE towards the poor resonates with previous studies using the CTP methods [35]. However, the CHE gap in our study between the poorest and the richest households was wider (i.e., 10:1) compared with 1.5:1 in Ibukun & Adebayo (2021). This is because of the methodological flaws in the budget share and WHO standard methods, which tend to overestimate the financial burden among wealthier households [17]. Our study also found that the cost of NCD medications was an important driver of CHE among NCD-affected households. This finding agrees with previous studies from LMIC [22, 62]. Studies have shown that NCD drugs are generally unavailable and unaffordable, particularly in Nigeria’s public facilities and pharmacies [63–65].

There was heterogeneity in CHE and impoverishment across states and geographical regions in the country. The concentration of CHE and impoverishment among NCD-affected households in rural and North East geopolitical locations, and Ebonyi, Sokoto, Adamawa, Zamfara, and Jigawa states was most likely due to a high prevalence of NCDs and their risk factors [66], limited health care access, illiteracy and poverty in these areas [45]. Arms conflict with a proliferation of internally displaced persons in the North East, perpetuating poverty and limiting health access, had some share in the blame [67]. Our finding that about 20% of NCD-affected households were impoverished or further impoverished by OOP payment aligns with the 25% estimated by Ibukun & Adebayo (2021) [13]. We also found that another 10% of households were on the verge of impoverishment after OOP payment. This is consistent with a Bangladesh study which, using a similar methodology, found 6.4–9.4% between 2005 and 2016 [68]. This study, like our study, also reported a negligible rate of unmet needs among households with NCDs. However, the rate was higher at the individual level. This lower household rate was most likely due to intra-household priority given to members with NCDs, who are more prone to severe complications and worsening costs if care is forgone. NCDs and poverty are like Siamese twins [9].

Studies have shown high indirect losses due to NCDs in LMICs [22]. We also found that NCD care was accompanied by substantial time and productivity losses. NCDs cost an individual an average of 58.51 lost workdays per year or 4.88 monthly. This finding reflects the results of hospital-based studies in Nigeria, which found workday losses of 5.3 and 8.56 per month among people with NCDs [13, 25]. Additionally, these studies estimated between 15 and 18.9% of income loss to NCDs’ in Nigeria, which agrees with our study’s estimate of about 10%. These results again echo previous studies [69, 70].

Our study has some limitations, mainly relating to the survey data used. The data were self-reported and could have introduced recall biases into our study, particularly regarding reporting NCDs, health expenditure and income. Moreover, considering the non-specific way diseases, especially NCDs, were named in the survey, there is a high possibility of misclassification errors. The above errors could have led to an overestimation or underestimation in our study. However, we gave due diligence to ensure accuracy wherever possible. The expunged data from Borno State, out of 37 states, due to non-representativeness, cannot significantly affect the generalizability of our result. The study is cross-sectional in nature and does not provide information on the long-term household economic effects of NCDs in Nigeria. This is especially important for chronic conditions like NCDs requiring regular prolonged care. Despite these drawbacks, the validity and applicability of our study’s findings provide valuable insight into the economic burden of NCDs in Nigeria.

The study has significant policy and practice implications in Nigeria. First, the high prevalence of NCDs and the associated economic burden, particularly among the poorest households, underscores the importance of addressing NCDs as a public health priority. This should be addressed by utilising targeted indigenous NCD prevention policies and policy equity, such as the National Multisectoral Action Plan (NNMSAP) for the Prevention and Control of NCDs, adapted from the WHO Global Action Plan on NCDs prevention [71, 72]. For these programmes to benefit those who most need them, they should be integrated into primary health care (PHC) and decentralised at the subnational level [73, 74]. Second, the high out-of-pocket costs associated with NCDs, especially for medication, accentuate the need for universal health coverage for vulnerable individuals through improved public-sector spending on health services, particularly for financially-intensive NCDs such as cancers, chronic renal diseases, and mental illness, and implementation of healthcare financing policy such as Nigeria’s new mandatory health insurance to improve availability, accessibility, and affordability [75, 76]. Third, the high concentration of CHE among the poor, rural households, and households in certain geographic regions has grave implications for health outcomes and health equity, highlighting the compelling need for improved awareness and bottom-up, targeted interventions among these populations [73, 77]. The possibility for impoverished households and those on the verge of poverty to fall into abject poverty underscores the need for policies and programmes that combat poverty and economic inequality. This could be accomplished by targeting the poor with social income transfer programmes that have been found to improve access to care for certain illnesses [78, 79]. Studies have shown that NCD admissions are increasing in Nigeria [80]. Coupled with vulnerability to physical, cognitive and psychological disabilities among people with NCDs, those policies required to prevent complications and disabilities among people with NCDs are obligatory. This study emphasises the need for a holistic approach that addresses the numerous causes of non-communicable diseases, such as poverty, access to health care, lifestyle, and environmental variables.

## Conclusion

This study contributes to our understanding of the cost of disease and studies on financial protection. It provides valuable insights into the economic burden of high OOP expenditure on NCDs, producing significant CHE, poverty, and productivity loss in Nigeria. As a crucial public health problem, particularly among poor and rural households in Nigeria, these issues require NCD prevention policies and universal health coverage at national and subnational levels to protect vulnerable households from financial hardship, poverty and health inequalities occasioned by NCDs. Future studies should continue to track the economic burden of NCDs, using a newly developed methodology and research instruments to analyse the effect of OOP spending and productivity loss on NCD-affected households. This may enable Nigeria to achieve the health-related Sustainable Development Goals (SDGs) by 2030 and help lift many out of poverty following the post-COVID-19 economic downturn.

### Supplementary Information

**Additional file 1: Supplementary Table 1.** Noncommunicable Disease Classification in the Study. **Supplementary Table 2.** Incidence of Catastrophic Health Expenditure (WHO Standard and Budget Share Methods) at Various Thresholds in Nigeria, 2018-19. **Supplementary Table 3.** Prevalence of Noncommunicable Diseases and Their Distribution across Age group, Gender and Rural-Urban Locations in Nigeria, 2018-19. **Supplementary Table 4.** Noncommunicable Disease-affected Households in Nigeria by Household Consumption Expenditure Quintile, 2018-19. **Supplementary Table 5.** Mean Household Spending of Non-communicable Disease-Affected Households in Nigeria, 2018-19. **Supplementary Table 6.** Mean per Capita Household Out-of-pocket Spending Compared with Total per Capita Heath Spending in Nigeria, 2018-19. **Supplementary Table 7.** Mean Per capita Share of Out-of-pocket Spending by Facility Type in Nigeria, 2018-19. **Supplementary Table 8.** Household Out-of-pocket Spending as a Share (%) of Total Household Consumption by Quintile in Nigeria, 2018-19. **Supplementary Table 9.** Incidence of Catastrophic Health Expenditure (WHO Standard and Budget Share methods) among Noncommunicable Disease-Affected Households in Nigeria by Socioeconomic Status, 2018-19. **Supplementary Table10.** Mean Catastrophic Health Expenditure at 40% for Noncommunicable Disease-Affected Households in Nigeria by Socioeconomic Status across Rural/Urban Locations, 2018-19. **Supplementary Table11.** Out-of-Pocket Spending by Households Experiencing Catastrophic Health Spending on Noncommunicable Diseases at 40% Threshold, 2018-19. **Supplementary Table 12.** Mean Catastrophic Health Expenditure at 40% for Noncommunicable Diseases-Affected Households by Geopolitical Zones in Nigeria, 2018-19. **Supplementary Figure 1.** Mean Annual Out-of-pocket Spending (Naira) by Individuals with Noncommunicable Disease, 2018-19. *Note*: Medicines include over-the-counter and patent drugs (including drugs during inpatient admission). Outpatient services include diagnostics (excluding drugs). Inpatient denotes costs of hospitalisation (excluding consultation fees and cost of medicines). **Supplementary Figure 2.** Incidence of Impoverishment and Further Impoverishment (%) among Noncommunicable Disease-Affected Households across States of Nigeria, 2018-19. **Supplementary Figure 3.** Mean Duration of Missed Primary Activity (in Days) Due to Poor Health from Noncommunicable Diseases in Nigeria, 2018-19. **Supplementary Figure 4.** Mean Duration of Inpatient Stays (in Days) by Noncommunicable Disease Type in Nigeria, 2018-19.

## Data Availability

The data supporting this study’s findings are available in the World Bank microdata library at https://microdata.worldbank.org/index.php/catalog/3827/get-microdata.
