# Supplementary material for: Economic burden of non-communicable diseases on households in Nigeria: evidence from the Nigeria living standard survey 2018-19
Source: BMC Public Health. 2023 Aug 17;23:1563. doi: 10.1186/s12889-023-16498-7 (PMC10433548; doi:10.1186/s12889-023-16498-7)
Supplement: Supplementary file 1 — Additional file 1: Supplementary Table 1. Noncommunicable Disease Classification in the Study. Supplementary Table 2. Incidence of Catastrophic Health Expenditure (WHO Standard and Budget Share Methods) at Various Thresholds in Nigeria, 2018-19. Supplementary Table 3. Prevalence of Noncommunicable Diseases and Their Distribution across Age group, Gender and Rural-Urban Locations in Nigeria, 2018-19. Supplementary Table 4. Noncommunicable Disease-affected Households in Nigeria by Household Consumption Expenditure Quintile, 2018-19. Supplementary Table 5. Mean Household Spending of Non-communicable Disease-Affected Households in Nigeria, 2018-19. Supplementary Table 6. Mean per Capita Household Out-of-pocket Spending Compared with Total per Capita Heath Spending in Nigeria, 2018-19. Supplementary Table 7. Mean Per capita Share of Out-of-pocket Spending by Facility Type in Nigeria, 2018-19. Supplementary Table 8. Household Out-of-pocket Spending as a Share (%) of Total Household Consumption by Quintile in Nigeria, 2018-19. Supplementary Table 9. Incidence of Catastrophic Health Expenditure (WHO Standard and Budget Share methods) among Noncommunicable Disease-Affected Households in Nigeria by Socioeconomic Status, 2018-19. Supplementary Table10. Mean Catastrophic Health Expenditure at 40% for Noncommunicable Disease-Affected Households in Nigeria by Socioeconomic Status across Rural/Urban Locations, 2018-19. Supplementary Table11. Out-of-Pocket Spending by Households Experiencing Catastrophic Health Spending on Noncommunicable Diseases at 40% Threshold, 2018-19. Supplementary Table 12. Mean Catastrophic Health Expenditure at 40% for Noncommunicable Diseases-Affected Households by Geopolitical Zones in Nigeria, 2018-19. Supplementary Figure 1. Mean Annual Out-of-pocket Spending (Naira) by Individuals with Noncommunicable Disease, 2018-19. Note: Medicines include over-the-counter and patent drugs (including drugs during inpatient admission). Outpatient services include di [file 12889_2023_16498_MOESM1_ESM.docx]

**SUPPLEMENTARY TABLES AND FIGURES**

**Supplementary Table 1: Noncommunicable Disease Classification in the Study**

| **Serial Number** | **Noncommunicable Diseases Group** | **^§^Specific Conditions in the Survey** |
| --- | --- | --- |
|  | Cardiovascular diseases | Hypertension, high blood pressure, strokes, ^*^heart problems, ^*^heartbeat problem, heart pain, cardiomegaly (enlarged heart) |
|  | Cancers (neoplasms) | Cancer, breast cancer (lump), leg cancer, prostate cancer, scrotal growth, neck tumour |
|  | Chronic respiratory diseases | Asthma, ^*^respiratory problems, |
|  | Diabetes | Diabetes |
|  | Mental disorders | ^*^Mental problem (illness), depression, memory loss |
|  | Neurological disorders | Faecal/urinary Incontinence, paralysis, seizure disorders (Epilepsy), Parkinson’s disease |
|  | Haematological disorders | Sickle cell diseases |
|  | Sense organ disorders | Visual impairment, ^*^eye problems, ^*^ear problems, cataracts |
|  | Renal (kidney) diseases | ^*^Kidney problems |
|  | Gastroenterological (digestive) diseases | Ulcers, appendicitis, heartburn, haemorrhoids, liver diseases, hernias, ^*^stomach problems, |
|  | Musculoskeletal disorders | Rheumatoid arthritis, Osteoarthritis, spondylosis, Gout, Waist pain, joint pain, rib pain, neck pain, low back pain, bone deformity, |
|  | Dermatological (skin and subcutaneous) diseases | Pruritus (body itching), body rashes, ^*^skin disease/problem |
|  | Dental (oral) diseases | Dental/^*^Teeth problem, toothache |
|  | Others Noncommunicable diseases (NCDs)  [urinary disorders, gynaecological diseases, and endocrine disorders] | ^*^Bladder problem, fibroid, goitre, prostate enlargement, ^*^menstrual problems, abortion/miscarriage, ^*^prostate problem, ^*^urinary problem, |
| *Note*: ^§^All descriptive or non-standard names (e.g. one-sided paralysis, madness, insanity) were replaced with their standard or medical names. ^*^However, we retained the descriptive group names in the form of “- problem” (e.g. mental problem) | | |

**Supplementary Table 2: Incidence of Catastrophic Health Expenditure (WHO Standard and Budget Share Methods) at Various Thresholds in Nigeria, 2018-19**

| **Household Type** | **WHO Standard Method**  **(**95%CI**) ^β^** | | | | | **Budget share method**  **(**95%CI**) ^β^** | | | |
| --- | --- | --- | --- | --- | --- | --- | --- | --- | --- |
|  | Threshold | | | | | Threshold | | | |
|  | 20% | 25% | 30% | 35% | 40% | 10% | 15% | 20% | 25% |
| **Households with NCDs**  ***n* [%]** | 1,931  [40.41] (38.51 – 42.32) | 1,505  [31.30] (29.46 – 33.13) | 1,150  [23.91] (22.24 – 25.58) | 897  [18.35] (16.86 – 19.83) | 677  [13.80] (12.45 – 15.15) | 1,878  [41.04] (39.06 – 43.01) | 1,167  [30.00] (24.22 – 27.77) | 691  [15.85] (14.36 – 17.34) | 430  [9.57] (8.46 –10.68) |
| **All households^*^**  ***n* [%]** | 4,884  [20.00] (19.08 – 20.93) | 3,643  [14.80] (14.00 – 15.59) | 2,715  [11.01] (10.34 – 11.69) | 2,045  [8.14] (7.59 – 08.70) | 1,533  [6.02] (5.56 – 6.47) | 4,460  [19.31] (18.48 – 20.15) | 2,459  [10.80] (10.17 – 11.43) | 1,380  [6.22] (5.75 –  6.69) | 818  [3.62] (3.27 –3.98) |
| Notes: ^*^Estimate for OOP spending on all health conditions, including NCDs and injuries.  ^β^ 95%CIs in round parenthesis underneath each estimate of catastrophic health expenditure. | | | | | | | | | |

**Supplementary Table 3: Prevalence of Noncommunicable Diseases and Their Distribution across Age group, Gender and Rural-Urban Locations in Nigeria, 2018-19**

| **Health Conditions** | **Total^β^** | **Age group^β^** | | | | **Gender^β^** | | **Location^β^** | |
| --- | --- | --- | --- | --- | --- | --- | --- | --- | --- |
|  | **Overall**  **(n= 5,653)** | **Children (5-18 years)** | **Young adults (19-34 years)** | **Middle-aged adults (35-56 years)** | **Elderly (Over 65 years)** | **Female**  **(n= 3,448)** | **Male**  **(n= 2,205)** | **Rural**  **(n= 4,230)** | **Urban**  **(n= 1,423)** |
| **All NCDs** | 16.80  (16.40 – 17.20) | 10.34  (9.80 – 10.91) | 41.42  (39.31 – 43.60) | 16.19  (15.30 – 17.12) | 32.80  (31.14 – 34.52) | 60.99  (59.72 – 62.26) | 39.01  (37.74 – 40.28) | 74.83  (73.68 – 75.94) | 25.17  (24.06 – 26.32 ) |
| **Cardiovascular Diseases** | 14.97  (14.06 – 15.92) | 0.91 (0.50 –1.63) | 3.19  (2.35 – 4.33) | 43.70  (39.18 –65.60) | 30.45  (27.64 –33.42) | 66.90  (63.66 – 69.99) | 33.10  (30.01 – 36.34) | 69.03  (65.83 – 72.06) | 30.97  (27.94 – 34.17) |
| **Cancers** | 0.30  (0.19 – 0.48) | 0.08  (0.01 – 0.58) | 0.32  (0.12 – 0.85) | 0.80  (0.32 – 2.01) | 0.31  (0.10 – 0.95) | 52.94  (30.27 – 74.46) | 47.06  (25.54 – 69.73) | 76.47  (51.45 – 90.88) | 23.53  (9.12 – 48.55) |
| **Respiratory Diseases** | 0.88  (0.67 – 1.17) | 0.74  (0.39 – 1.42) | 0.88  (0.49 – 1.58) | 1.85  (1.00 –3.40) | 1.03  (0.55 – 1.90) | 62.00  (47.96 – 74.28) | 38.00  (25.72 – 52.04) | 60.00  (46.00 – 72.54) | 40.00  (27.46 – 54.00) |
| **Diabetes** | 2.90  (2.49 – 3.37) | 0.17  (0.04 – 0.66) | 0.56  (0.27 – 1.17) | 7.66  (5.82 –10.16) | 6.79  (5.37 – 8.55) | 46.95  (39.44 – 54.60) | 53.05  (45.40 – 60.56) | 55.49  (47.81 – 62.91) | 44.51  (37.09 – 52.19) |
| **Mental Disorders** | 0.64  (0.46 – 0.88) | 0.41  (0.17 – 0.99) | 1.28  (0.78 – 2.08) | 1.25  (0.64 –2.60) | 0.21  (0.05 – 0.82) | 44.44  (29.31 – 60.69) | 55.56  (39.31 – 70.69) | 72.22  (55.63 – 84.35) | 27.78  (15.65 – 44.37) |
| **Neurological Diseases** | 0.50  (0.34 – 0.72) | 1.07  (0.62 – 1.84) | 0.72  (0.37 – 1.38) | 0.35  (0.09 –1.47 ) | 0.21  (0.05 – 0.82) | 57.14  (38.68 – 73.81) | 42.86  (26.19 – 61.32) | 75.00  (56.05 – 87.59) | 25.00  (12.41 – 43.95) |
| **Haematological Diseases** | 0.28  (0.17 – 0.46) | 0.99  (0.56 –1.74) | 0.32  (0.12 – 0.85) | 0.00 | 0.00 | 37.50  (17.90 – 62.28) | 62.50  (37.72 – 82.10) | 56.25  (32.38 – 77.54) | 43.75  (22.46 – 67.62) |
| **Sense Organ Diseases** | 9.71  (8.97 – 10.51) | 10.57  (8.96 – 12.43) | 4.95  (3.88 – 6.30) | 15.75  (12.87 – 19.22) | 18.72  (16.39 – 21.30) | 52.46  (48.27 – 56.61) | 47.54  (43.39 – 51.73) | 75.41  (71.63 – 78.83) | 24.59  (21.17 –28.37) |
| **Renal Diseases** | 0.19  (0.11 – 0.35) | 0.33  (0.12 –0.88) | 0.24  (0.08 –0.74) | 0.17  (0.04 – 0.66) | 0.21  (0.05 – 0.82) | 45.45  (20.28 – 73.19) | 54.55  (26.81 – 79.72) | 81.82  (49.30 – 95.42) | 18.18  (4.58 – 50.70) |
| **Gastroenterological Diseases** | 51.69  (50.39 – 52.99) | 63.58  (60.83 –66.25) | 74.52  (72.03 – 76.86) | 92.2  (86.58 –98.03 | 22.33  (19.82 –25.05) | 63.31  (61.55 – 65.04) | 36.69  (34.96 – 38.45) | 78.20  (76.67 – 79.66) | 21.80  (20.34 – 23.33) |
| **Musculoskeletal Diseases** | 3.34  (2.91 – 3.85) | 1.98  (1.33 – 2.94) | 1.68  (1.10 –2.56) | 8.27 (6.13 –10.84) | 5.45  (4.19 – 7.07) | 58.20  (51.05 – 65.02) | 41.80  (34.98 – 48.95) | 71.43  (64.58 – 77.41) | 28.57  (22.59 – 35.42) |
| **Dermatological Diseases** | 5.02  (4.48 – 5.62) | 12.8  (11.11 – 14.89) | 4.39  (3.39 – 5.68) | 5.60 (3.96 – 7.92) | 1.23  (0.70 – 2.16) | 47.18  (41.44 – 53.00) | 52.82  (47.00 – 58.56) | 75.00  (69.64 – 79.69) | 25.00  (20.31 – 30.36) |
| **Dental Diseases** | 4.62  (4.10 – 5.20) | 5.45  (4.30 –6.88) | 4.55  (3.53 –5.86) | 10.95 (8.58 –13.94 | 4.08  (3.09 –5.35) | 60.54  (54.48 – 66.29) | 39.46  (33.71 – 45.52) | 75.48  (69.90 – 80.32) | 24.52  (19.68 – 30.10) |
| **Other NCDs** | 0.58  (0.42 – 0.82) | 0.08  (0.01 –0.58) | 1.0  (0.60 –1.78) | 1.40 (0.70 – 2.82) | 0.41  (0.15 – 1.09) | 66.67  (49.23 – 80.49) | 33.33  (19.51 – 50.77) | 72.73  (55.35 – 85.16) | 27.27  (14.84 – 44.65) |
| **NCD Comorbidity** | 4.37  (3.87 – 4.93) | 0.74  (0.39 – 1.42) | 1.36  (0.85 – 2.17) | 10.55 (8.33 – 13.39) | 10.19  (8.43 – 12.25) | 64.78  (58.62 – 70.48) | 35.22  (29.52 – 41.38) | 72.47  (66.57 – 77.68) | 27.53  (22.32 – 33.43) |
| *Note*: ^β^95% CIs in parenthesis in parenthesis underneath each estimate. | | | | | | | | | |

**Supplementary Table 4: Noncommunicable Disease-affected Households in Nigeria by Household Consumption Expenditure Quintile, 2018-19**

| **Households by Consumption Expenditure Quintile** | **Prevalence in percent ^β^** |
| --- | --- |
| poorest  *n* [%] | 1,187  [21.01]  (19.35 – 22.76) |
| poor  *n* [%] | 1,121  [22.36]  (21.00 – 23.79) |
| Middle  *n* [%] | 953  [21.31]  (19.99 – 22.70) |
| rich  *n* [%] | 755  [19.03]  (17.65 – 20.48) |
| Richest  *n* [%] | 544  [16.29]  (14.50 – 18.25) |
| *Note*: ^β^95% CIs in round parenthesis underneath each estimate | |

**Supplementary Table 5:Mean Household Spending of Non-communicable Disease-Affected Households in Nigeria, 2018-19**

| **Mean Household Expenditure (₦)** | | |  |
| --- | --- | --- | --- |
| **Consumption Quintile** | **out-of-pocket** | **Food** | **Total** |
| Poorest | 57615.07  (52950.20–62279.95) | 322502.10  (307294.70–337709.50) | 556986.60  (532450.40–581522.90) |
| Poor | 78682.62  (73348.26–84016.97) | 448123.70  (430594.10–465653.20) | 825919.90  (798956.50–852883.40) |
| Middle | 111880.5  (103260.50–120500.50) | 523534.60  (498686.00–548383.20) | 1027495.00  (987299.60–1067691.00) |
| Rich | 139190.3  (127343.30–151037.30) | 602776.60  (572096.60–633456.50) | 1289311.00  (1233618.00–1345004.00) |
| Richest | 259572.1  (195826.10–323318.10) | 682917.20  (627605.40–738229.00) | 1904266.00  (1755971.00–2052561.00) |
| **Total** | 122313.60  (111005.70–133621.50) | 505483.40  (489979.30–520987.50) | 1076230.00  (1038165.00–1114295.00) |
| ***Note:*** ^β^95% CIs in parenthesis in parenthesis underneath each estimate. | | | |

**Supplementary Table 6: Mean per Capita Household Out-of-pocket Spending Compared with Total per Capita Heath Spending in Nigeria, 2018-19**

| **Health conditions** | **Mean out-of-pocket spending (₦)^β^** | **Mean total health spending (₦)^β^** |
| --- | --- | --- |
| **Non-communicable diseases** | 20092.44 (18158.81 – 22026.08) | 21387.82 (19426.08 – 23349.56) |
| **All conditions**^§^ | 11151.16 (10545.88 – 11756.45) | 11839.15 (11220.15 – 12458.14) |
| *Note:*^§^All conditions denote all illnesses, including NCDs (noncommunicable diseases) and injuries.  ^β^95% CIs in parenthesis in parenthesis underneath each estimate. | | |

**Supplementary Table 7: Mean Per capita Share of Out-of-pocket Spending by Facility Type in Nigeria, 2018-19**

| **Health Conditions** | **Mean out-of-pocket spending (₦)^β^** | | |
| --- | --- | --- | --- |
|  | **Private facility** | **Public facility** | **Combined** |
| **Non-communicable diseases** | 93660.85  (83026.36 – 104295.3) | 68884.07  (61494.89 – 76273.26) | 52409.98  (48860.91 – 55959.06) |
| **All conditions**^§^ | 49660.34  (47339.42 – 51981.27) | 42246.98  (40222.56 – 44271.4) | 10752.17  (10423.49 – 11080.85) |
| *Note:*^§^All conditions denote all illnesses, including noncommunicable diseases and injuries.  ^β^95% CIs in parenthesis in parenthesis underneath each estimate. | | | |

**Supplementary Table 8: Household Out-of-pocket Spending as a Share (%) of Total Household Consumption by Quintile in Nigeria, 2018-19**

| **Households by Consumption Expenditure Quintile** | **Budget share of Consumption** |
| --- | --- |
| **poorest** | 10.34 (9.56 – 11.12) |
| **poor** | 9.53 (8.87 – 10.18) |
| **middle** | 10.89 (10.08 – 11.69) |
| **rich** | 10.80 (9.93 – 11.66) |
| **richest** | 13.63 (10.62 – 16.64) |
| ***Note:*** ^β^95% CIs in parenthesis in parenthesis underneath each estimate. | |

**Supplementary Table 9: Incidence of Catastrophic Health Expenditure (WHO Standard and Budget Share methods) among Noncommunicable Disease-Affected Households in Nigeria by Socioeconomic Status, 2018-19**

| **Households by Consumption Expenditure Quintile** | **Mean Catastrophic Health Expenditure (%)** | |
| --- | --- | --- |
|  | **WHO standard method, 40% threshold (95%CI)^β^** | **Budget share method, 25% threshold (95%Cl)^β^** |
| **Poorest**  *n* [%] | 637  [20.31]  (17.35 – 23.27) | 193  [8.31]  (6.12 – 10.50) |
| **Poor**  *n* [%] | 379  [13.89]  (11.55 – 16.23) | 173  [7.11]  (5.38 – 08.83) |
| **Middle**  *n* [%] | 279  [13.84]  (11.33 – 16.35) | 189  [10.87]  (8.51 – 13.24) |
| **Rich**  *n* [%] | 143  [9.31]  (7.04 – 11.57) | 141  [9.32]  (7.06 – 11.58) |
| **Richest**  *n* [%] | 95  [10.49]  (07.45 – 13.53) | 122  [13.17]  (9.97 – 16.38) |
| *Notes*: ^β^ 95%CI in round parenthesis underneath each estimate of catastrophic health expenditure. | | |

**Supplementary Table 10: Mean Catastrophic Health Expenditure at 40% for Noncommunicable Disease-Affected Households in Nigeria by Socioeconomic Status across Rural/Urban Locations, 2018-19**

| **Percentage Mean Catastrophic Health Expenditure at 40% ^β^** | | | |
| --- | --- | --- | --- |
| **Household by consumption quintile** | **All households** | **Rural** | **Urban** |
| Poorest  *n* [%] | 970  [81.71]  (79.02 – 84.40) | 869  [82.40]  (79.62 – 85.19) | 101  [76.99]  (68.07 – 85.91) |
| Poor  *n* [%] | 281  [23.92]  (20.92 – 26.92) | 237  [24.76]  (21.41 – 28.11) | 44  [20.88]  (14.22 – 27.53) |
| Middle  *n* [%] | 132  [14.60]  (11.95 – 17.25) | 107  [15.90]  (12.78 – 19.01) | 25  [11.39]  (6.48 – 16.30) |
| Rich  *n* [%] | 70  [8.04]  (5.92 – 10.16) | 51  [10.57]  (7.38 – 13.75) | 19  [4.42]  (2.10 – 6.75) |
| Richest  *n* [%] | 52  [8.50]  (5.88 – 11.12) | 31  [12.50]  (7.96 – 17.05) | 21  [6.00]  (2.91 – 9.10) |
| **Total**  ***n* [%]** | 1,505  [28.54]  (26.69 – 30.39) | 1295  [34.79]  (32.56 – 37.01) | 210  [14.97]  (12.55 – 17.39) |
| *Note:* ^β^95% CIs are shown in round parenthesis underneath each estimate of catastrophic health expenditure. | | | |

**Supplementary Table 11: Out-of-Pocket Spending by Households Experiencing Catastrophic Health Spending on Noncommunicable Diseases at 40% Threshold, 2018-19**

| **Health Service Type**^*^ | **Socioeconomic Status** | | | | | **Total**  **(%)** |
| --- | --- | --- | --- | --- | --- | --- |
|  | **Poorest**  **(%)** | **Poor**  **(%)** | **Middle**  **(%)** | **Rich**  **(%)** | **Richest**  **(%)** |  |
| Medicine | 91.82 | 88.21 | 89.80 | 86.04 | 83.21 | 87.32 |
| Outpatient | 4.53 | 6.16 | 4.77 | 4.12 | 8.86 | 6.25 |
| Inpatient | 3.65 | 5.63 | 5.44 | 9.84 | 7.93 | 6.43 |
| *Notes*: The households were grouped according to consumption quintiles.  ^*^Medicines include over-the-counter and patent drugs (including drugs during inpatient admission). Outpatient services include diagnostics (excluding drugs). Inpatient denotes costs of hospitalisation (excluding consultation fees and cost of medicines). | | | | | | |

**Supplementary Table 12: Mean Catastrophic Health Expenditure at 40% for Noncommunicable Diseases-Affected Households by Geopolitical Zones in Nigeria, 2018-19**

| **Geopolitical Zone** | **Percentage Mean Catastrophic Health Expenditure at 40% ^β^** |
| --- | --- |
| North Central  *n* [%] | 237  [16.36]  (15.24 – 17.48) |
| North East  *n* [%] | 455  [39.40]  (37.65 – 41.15) |
| North West  *n* [%] | 402  [34.56]  (33.12 – 36.00) |
| South East  *n* [%] | 274  [27.38]  (25.78 – 28.97) |
| South South  *n* [%] | 85  [6.98]  (6.14 – 7.81) |
| South West  *n* [%] | 52  [4.98]  (4.27 – 5.69) |
| *Note:* ^β^95% CIs are shown in round parenthesis underneath each estimate of catastrophic health expenditure. | |


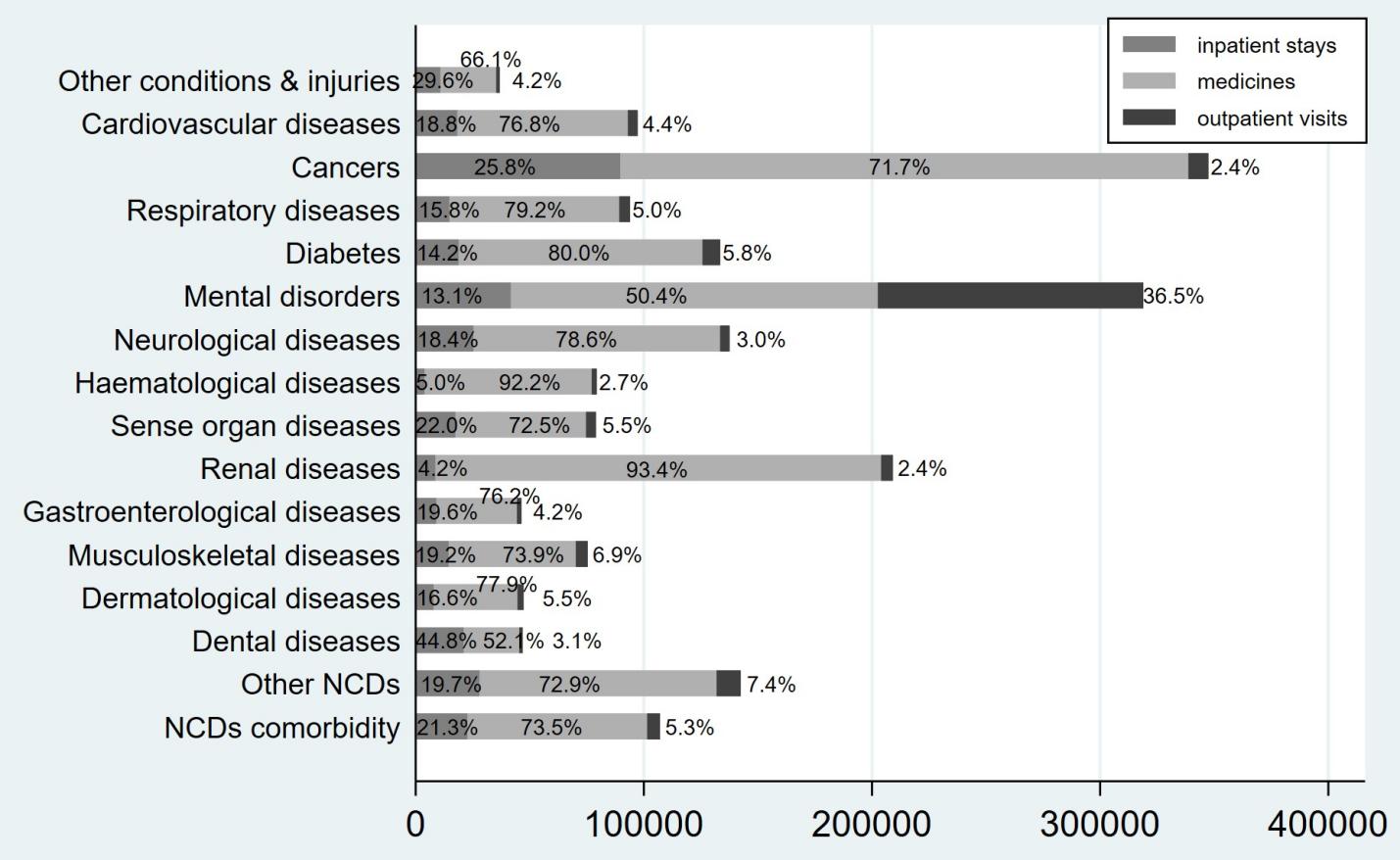


**Supplementary Figure 1: Mean Annual Out-of-pocket Spending (Naira) by Individuals with Noncommunicable Disease, 2018-19**

*Note*: Medicines include over-the-counter and patent drugs (including drugs during inpatient admission). Outpatient services include diagnostics (excluding drugs). Inpatient denotes costs of hospitalisation (excluding consultation fees and cost of medicines)


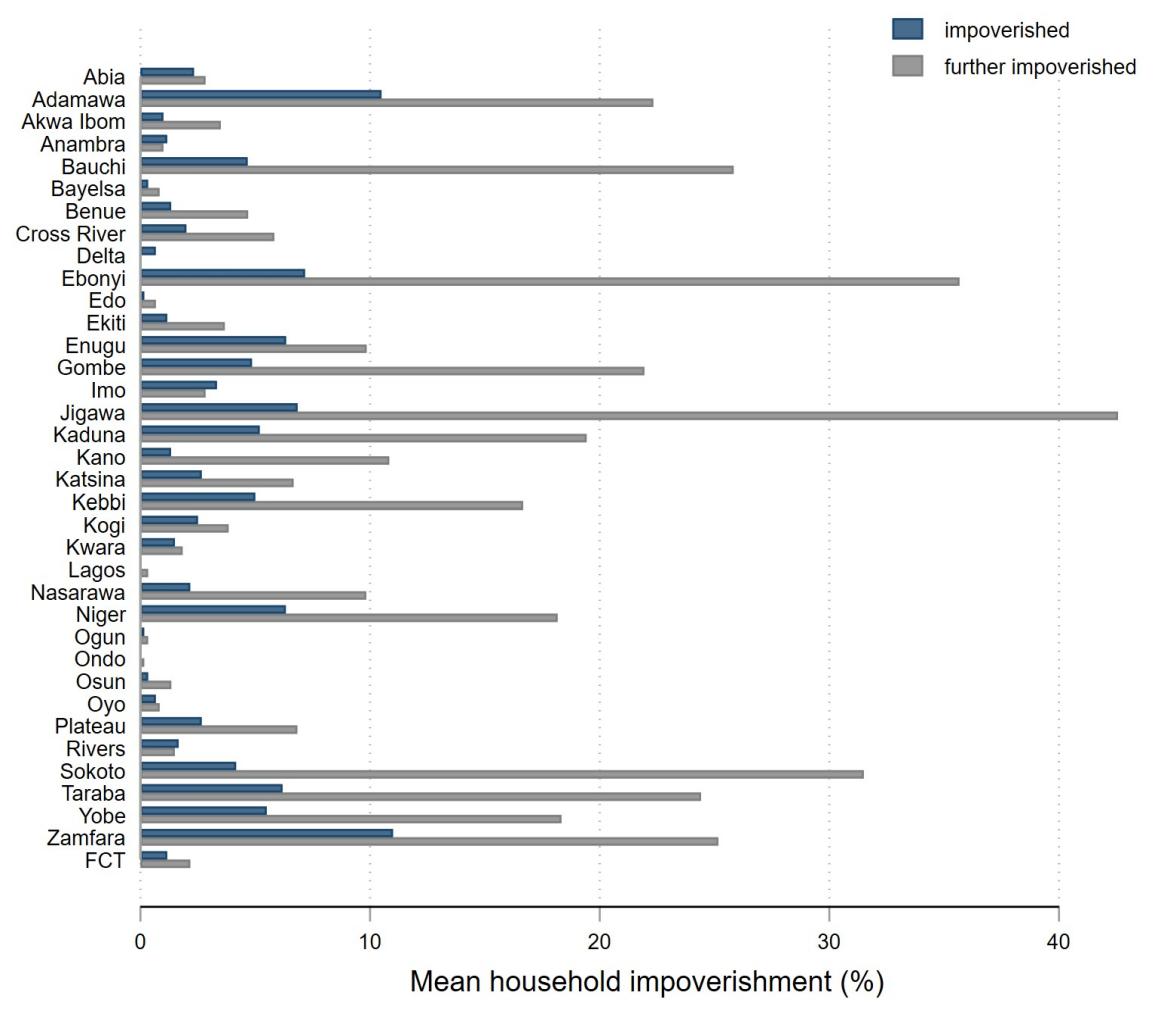


**Supplementary Figure 2: Incidence of Impoverishment and Further Impoverishment (%) among Noncommunicable Disease-Affected Households across States of Nigeria, 2018-19**


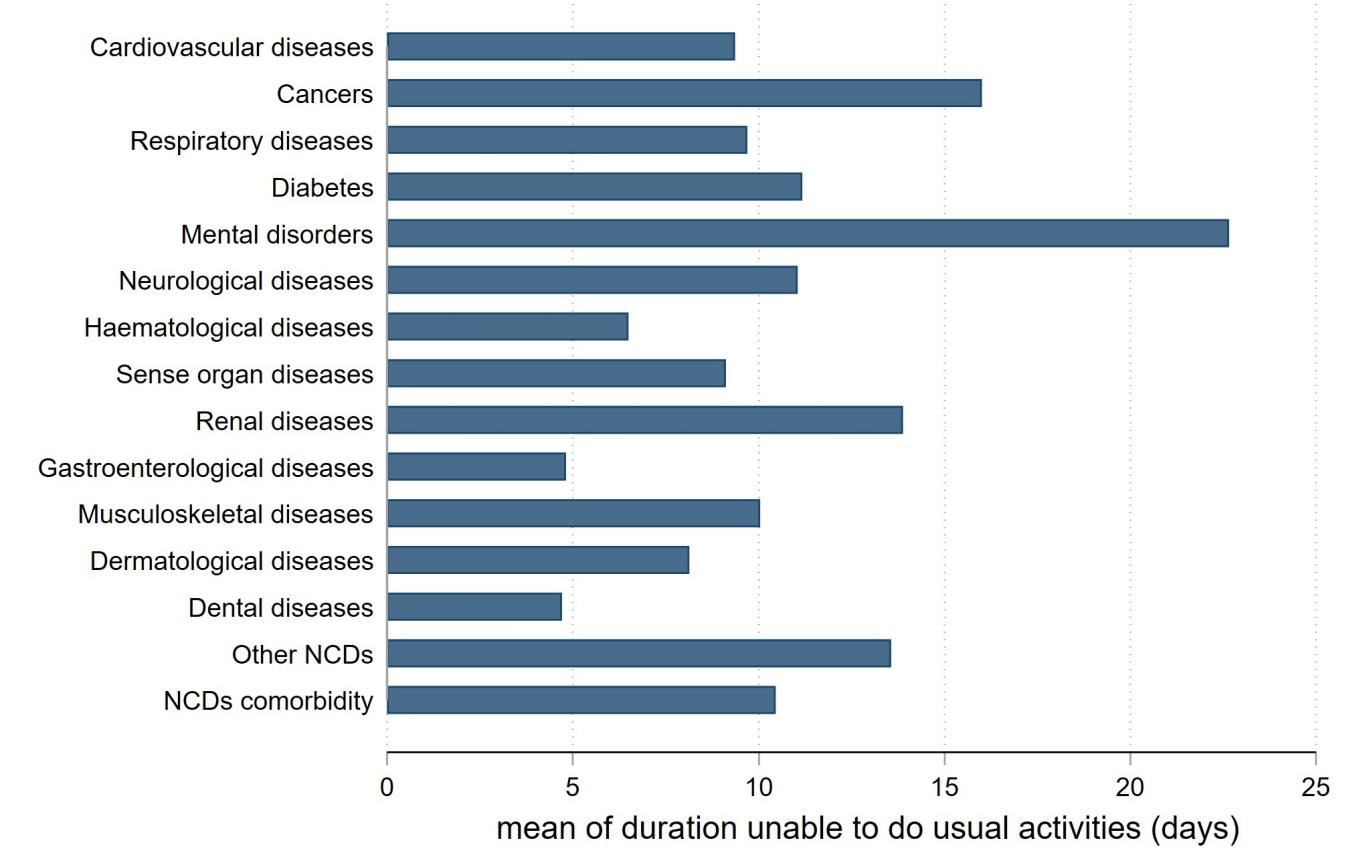
**Supplementary Figure 3: Mean Duration of Missed Primary Activity (in Days) Due to Poor Health from Noncommunicable Diseases in Nigeria, 2018-19**


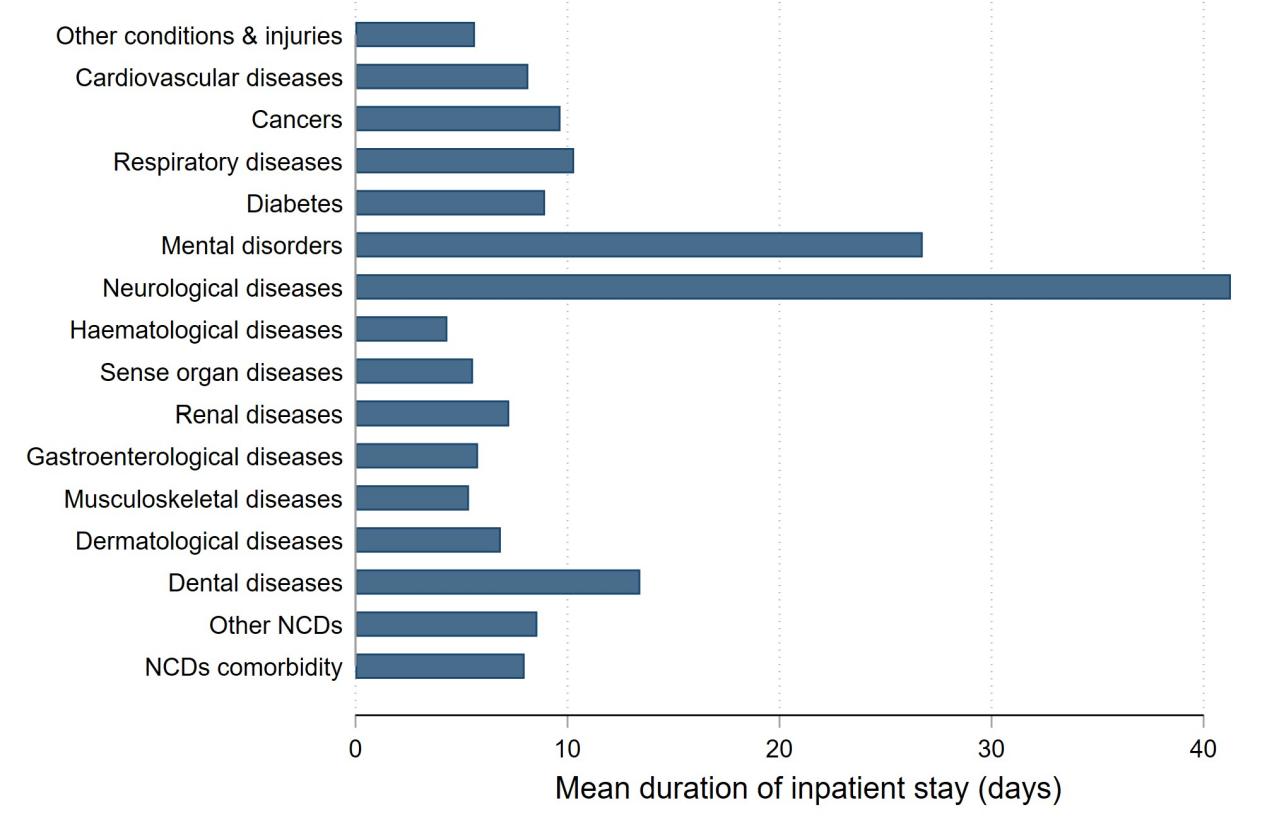


**Supplementary Figure 4: Mean Duration of Inpatient Stays (in Days) by Noncommunicable Disease Type in Nigeria, 2018-19**
